# Status of stem cells in diabetic nephropathy: predictive and preventive potentials

**DOI:** 10.1186/s13167-016-0070-6

**Published:** 2016-10-04

**Authors:** Babak Baban, Jun Yao Liu, Samuel Payne, Worku Abebe, Jack C. Yu, Mahmood S. Mozaffari

**Affiliations:** 1 Department of Oral Biology; CL-2140, Dental College of Georgia, Augusta University, Augusta, GA 30912-1128 USA; 2 Department of Surgery, Section of Plastic Surgery, Medical College of Georgia, Augusta, GA 30912 USA; 3 Department of Medicine, Medical College of Georgia, Augusta University, Augusta, GA 30912 USA

**Keywords:** Diabetic nephropathy, Stems cells, Blood, Kidney, Apoptosis, Predictive preventive and personalized medicine

## Abstract

**Background:**

Recruitment of stem cells to sites of tissue injury constitutes an important mechanism aimed at tissue repair and regeneration. However, it is not clear how the diabetic milieu affects the viability of endogenous stem cells. Thus, we tested the hypothesis that diabetes mellitus is associated with increased apoptosis which, in turn, contributes to reduction in stem cells and the manifestation of type 2 diabetic nephropathy.

**Methods:**

Sixteen-week-old male obese type 2 diabetic db/db mice, and their appropriate controls, were used for assessment of the status of endothelial progenitor cells (EPCs), mesenchymal stem cells (MSCs), and hematopoetic stem cells (HSCs) in the peripheral blood and renal tissue using specific cell markers. Further, we explored whether diabetic animals display greater apoptosis of stem cell subsets.

**Results:**

The peripheral blood cells of db/db mice displayed reduction in EPCs (*p* < 0.05) compared to those of db/m controls. Further, kidney cells prepared from experimental groups also showed reductions in EPCs, MSCs, and HSCs. We also observed increased apoptosis of stem cell subsets in cells prepared from kidneys of db/db than those of db/m mice.

**Conclusions:**

The present study shows a similar pattern of decline in stem cell subsets in peripheral blood and kidneys of db/db mice, an effect likely related to increased apoptosis. Collectively, the results suggest that apoptosis of stem cells likely contributes to eventual manifestation of renal failure in diabetes mellitus. Monitoring of blood levels of stem cell subsets could predict failure of their reparative and protective effects and eventual manifestations of diabetic complications.

## Background

The worldwide epidemic of diabetes mellitus, and associated micro- and macro-angiopathic complications, is one of the most pressing health-related challenges of our time. Nephropathy is a major complication of diabetes mellitus with a prevalence of 25–40 % [1]. Diabetic nephropathy is characterized as glomerular and tubular dysfunctions and variable increase in urinary albumin excretion. The hallmark feature of diabetic nephropathy is the decline in the glomerular filtration rate which ultimately leads to end-stage renal disease (ESRD) thereby requiring renal replacement therapy [2, 3]. Indeed, 44 % of new cases of ESRD in the USA are caused by diabetic nephropathy [1, 4].

The American Diabetes Association considers strict glycemic control as the first-line intervention for the prevention of diabetic complications [2, 5]. While strict glycemic control delays the onset of diabetic nephropathy, it does not completely prevent its progression. Other intervention modalities include control of blood pressure and use of pharmacological agents targeting the renin-angiotensin-aldosterone axis. Nonetheless, clinical trials do not suggest that such intervention modalities prevent progression to ESRD [2, 6]. This suggests not only the inadequacy of current drug therapies but also the failure of endogenous protective and reparative mechanisms. This recognition has led to exploration of the potential usefulness of stem cells in beneficially influencing the course of diabetic complications.

The beneficial effects of bone marrow-derived stem cells and cord blood stems cells in the non-diabetic obese mouse (an animal model of type 1 diabetes mellitus) have been established. The rationale for the use of stem cells (e.g., in type 1 diabetes), in part, relates to their immunoregulatory properties and promotion of peripheral tolerance to pancreatic β cells [7]. Importantly, hematopoietic stem cells rescue peripheral tolerance towards pancreatic β cells in type 1 diabetic subjects raising the prospect for a stem cell-based treatment regimen to prevent or delay the development of chronic complications of the disease [8].

With respect to the kidney, in the streptozotocin-induced type 1 diabetic mice, mesenchymal stem cell (MSC) administration ameliorated histopathological features including fibrosis, glomerulosclerosis, glomerular basement thickening, capillary occlusion, reduced podocyte density, and effacement of foot processes. Authors concluded that, despite persistence of dysglycemia, MSC administration exerts renoprotection likely via the promotion of a pro-regenerative microenvironment [9]. Although the role of inflammation in type 2 diabetic nephropathy is increasingly appreciated [10], there is a relative lack of information regarding the usefulness of stem cells in this condition.

The beneficial effects of stem cells in tissue repair and regeneration are related primarily to the release of soluble factors which, in turn, curtail the inflammatory response to tissue injury. Importantly, however, the diabetic milieu exerts detrimental effects on the number and function of stem cells [11–15]. Given that the vast majority of diabetic patients have type 2 diabetes and type 2 diabetic nephropathy is the leading cause of end-stage renal diseases, it is essential to unravel the impact of diabetic milieu on stem cells in this disease condition. Thus, the present study was intended to explore the status of components of stem cells in the setting of type 2 diabetic nephropathy. Accordingly, we tested the hypothesis that apoptosis of stem cells contributes to impaired reparative/regenerative capacity in type 2 diabetic nephropathy. Ultimately, a more comprehensive dynamic and mechanistic understanding is needed combining experimental data with computational modeling so that a targeted therapeutic approach can be possible.

## Methods

Male db/m and db/db mice (*n* = 5/group) were purchased from the Jackson Laboratories and housed, with free access to food and fluid, in the laboratory animal facilities at Augusta University. The use of animals for these studies conformed to the institutional guidelines for the care and use of laboratory animals.

We have shown that hallmark features of db/db mice, compared to their lean controls, include (a) a significant increase in body weight, (b) a significant increase in homeostatic model assessment index, a measure of insulin resistance, (c) a significant increase in hemoglobin A1c, an index of chronic glycemic control, (d) a significant increase in albumin excretion but reduction in creatinine clearance, and (e) histopathological changes including mesangial expansion and glomerular sclerosis [16, 17]. Starting at about 8 weeks of age, these features become progressively more prominent, and by about 16 weeks of age, these hallmark features are fully manifested [16]. Given its hallmark features, the db/db mouse is a very relevant animal model of type 2 diabetic nephropathy.

At 16 weeks of age, db/db and db/m mice were sacrificed and blood samples were obtained for determination of peripheral blood stem cell subsets. Also, the kidneys were procured for the preparation of renal cells and assessment of subsets of stem cells and apoptosis. In addition, cytospins were prepared using renal cell preparations for subsequent immunofluorescent assessment of caspase 3-positive stem cell subsets. Identification of stem cell subsets was based on the use of specific cell markers as follows: hematopoetic stem cells (HSCs): Sca1^+^cKit^+^CD31^−^; mesenchymal stem cells (MSCs): Sca1^+^CD105^+^CD31^−^; and endothelial progenitor cells (EPCs): Sca1^+^ckit^+^CD31^+^ [18].

### Assessment of stem cells in peripheral blood and kidney

To identify and evaluate stem cells in renal tissue and blood, we employed a flow cytometry-based assay which is well-established in our lab [18]. Briefly, for renal stem cells, kidney samples were sieved through a cell strainer (BD Biosciences, San Diego, CA) followed by centrifugation (1500 rpm, 10 min) to prepare single-cell suspensions. For blood, samples of whole blood were treated with reagents and stained with fluorochrome-conjugated antibodies of interest based on the manufacturers’ instructions. Antibodies against Sca1, cKit, CD105, CD73, CD31 (markers of murine stem cells), and caspase 3 (for apoptosis) were obtained from BD Biosciences (San Diego, CA). Phenotypic analyses of stem cells were performed as described previously [19, 20]. Briefly, cells were run through a four-color flow cytometer (FACS Calibur, BD Biosciences, San Diego, CA), and data were collected using CellQuest software. Samples were double-stained with control IgG and cell markers and were used to assess any spillover signal of fluorochromes; proper compensation was set to ensure the median fluorescence intensities of negative and positive cells were identical and were both gated populations. Gating was used to exclude dead cells and debris using forward and side scatterplots. In each analysis, 100,000 total events were collected. As a gating strategy, for each sample, isotype-matched controls were analyzed to set the appropriate gates. For each marker, samples were analyzed in duplicate measurements. To minimize false-positive events, the number of double-positive events detected with the isotype controls was subtracted from the number of double-positive cells stained with corresponding antibodies (not isotype control), respectively. Cells expressing a specific marker were reported as a percentage of the number of gated events.

### Cytospin technique and immunofluorescence staining

To maximize the yield of cells from the specimen, cytospin technique was used as described previously [21]. Briefly, sorted cells per sample chamber (~20,000 cells) were centrifuged (700 rpm, 5 min), air-dried, fixed in 10 % formalin, and washed twice in PBS. All subsequent procedures were carried out at room temperature (RT). Endogenous peroxidase activity was blocked with hydrogen peroxide (1:10 w/PBS, 10 min). All slides were washed three times for 5 min at room temperature and then incubated in blocking buffer (20 % normal donkey serum, 1 % BSA, 0.02 % NaN_3_, 1× PBS) for 45–60 min. Following treatment with the primary antibodies overnight at 4 °C, preparations were then washed three times with Tris-buffered saline for 5 min each time. All slides were then incubated with the secondary fluorescence-labeled antibody (all secondary Abs were purchased from Jackson Immunoresearch Laboratories, West Grove, PA) for 1 h in the dark at room temperature, washed twice in Tris-buffered saline for 5 min each time in the dark, and then counterstained using 4′,6-diamidino-2-phenylindole (DAPI) nuclear staining, mounted, and were subjected to microscopic examination.

## Results

**Fig. 1:**
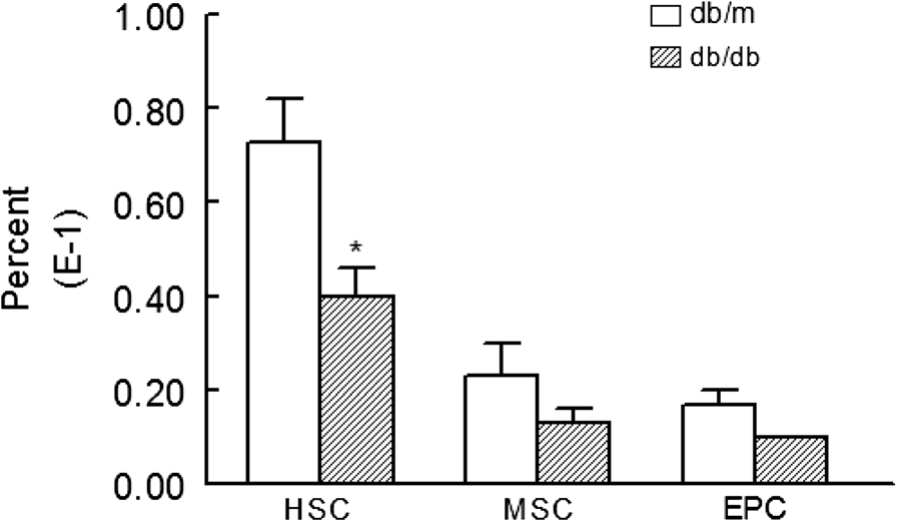
Bar graphs show levels of HSCs (Sca1^+^cKit^+^CD31^−^), MSCs (Sca1^+^CD105^+^CD31^−^), and EPCs (Sca1^+^ckit^+^CD31^+^) in peripheral blood of db/db and db/m mice. *p < 0.05 compared to the db/m group

**Fig. 2:**
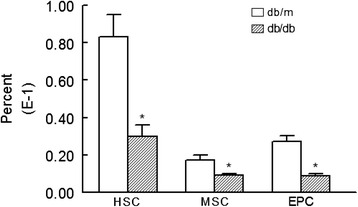
Bar graphs show kidney cells prepared from db/db mice display reduced levels of HSC, MSC, and EPC compared to their db/m controls. *p < 0.05 compared to the db/m group

Figure 1 shows percent of HSCs, MSCs, and EPCs (expressed as of total circulating cells in peripheral blood), using specific markers as described in the “Methods” section, in the peripheral blood of experimental groups. Although each stem cell subset was reduced in db/db mice compared to the db/m group, the difference between the two groups was significant for HSCs. On the other hand, Fig. 2 shows that cell preparations from kidneys of db/db mice have significant reduction in HSCs, MSCs, and EPCs compared to kidney cell preparations of db/m mice, and the reduction in HSC in db/db mice was even greater compared to that in peripheral blood (Fig. 1).

**Fig. 3:**
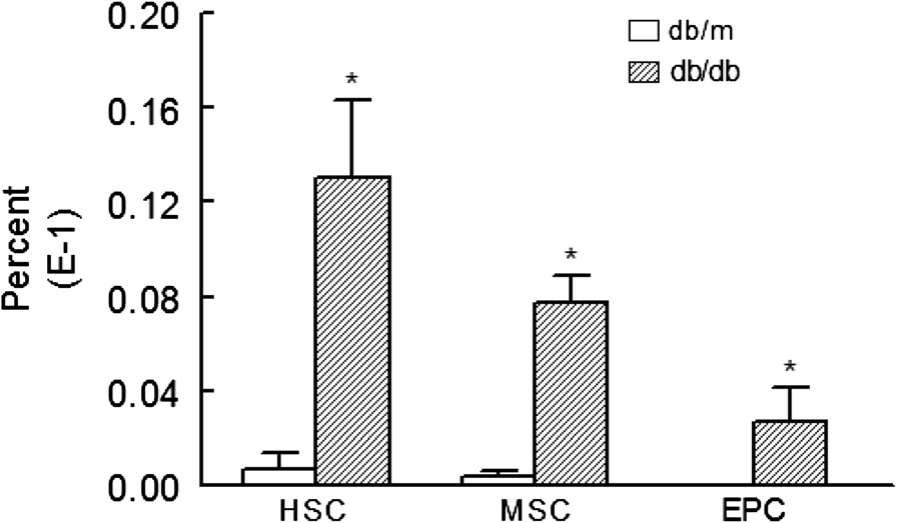
Bar graphs show increased apoptotic HSCs, MSCs, and EPCs in cell preparations obtained for kidneys of db/db than db/m mice. *p < 0.05 compared to the db/m group

**Fig. 4:**
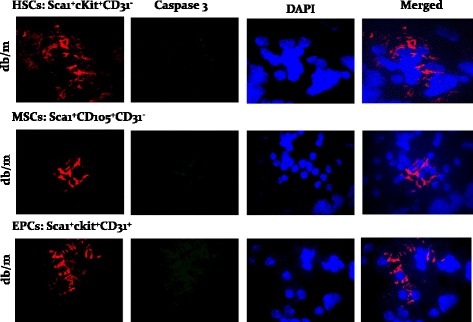
Panels show immunofluorescent images of stem cell subsets for caspase 3 and DAPI and their merged images, for db/m mice

**Fig. 5:**
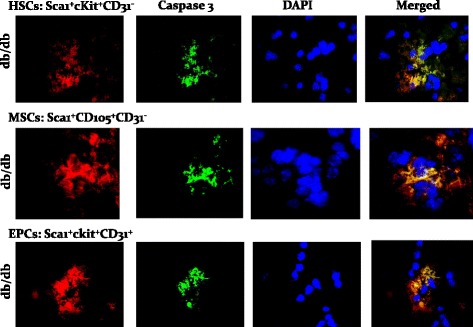
Panels show immunofluorescent images of stem cell subsets for caspase 3 and DAPI, and their merged images, for db/db mice

We conjectured that increased apoptosis likely contributes to reduction in stem cell subsets in cell preparations from kidneys of db/db mice compared to those of their lean db/m controls. As shown in Fig. 3, indeed, db/db mice displayed significant increase in apoptotic cell death in kidney stem cell subsets compared to db/m controls. To provide further support for our working hypothesis that the diabetic milieu increases susceptibility of stem cells to apoptosis, cytospin preparations of kidney cells were subjected to immunofluorescent staining for caspase 3 while DAPI was used as a nuclear marker; these studies were carried out in conjunction with the use of specific markers for each subset of stem cells. While there was no discernable apoptotic foci for the db/m control group (Fig. 4), stem cell subsets of db/db kidneys showed marked immunofluorescent staining for caspase 3 and merged images further confirm prominent apoptosis of stem cell subsets of db/db kidneys (Fig. 5).

## Discussion

The present study shows reductions in subsets of stem cells in peripheral blood and renal cell preparations of db/db mice, a model of type 2 diabetic nephropathy. We further show increased apoptosis of HSCs, MSCs, and EPCs in cell preparations from kidneys of db/db compared to db/m mice. Collectively, the results suggest that the diabetic milieu exerts detrimental effects on survivability of stem cell subsets in this animal model. Given that stem cells are pivotal players in tissue repair and regeneration following injury, reduction in their numbers (and/or function) impairs the ability of the organism to cope with injury thereby contributing to progressive worsening of renal architecture and dysfunction.

Diabetes is the most common cause of ESRD [2]. Thus, there is an intense interest in slowing/halting the progression of kidney disease and even promoting regression of renal lesions and associated kidney dysfunction. Indeed, remission of the disease and regression of renal lesions can occur in experimental animals and human subjects; regression of glomerular structural changes is associated with remodeling of the glomerular architecture [14]. In specific circumstances, renal injuries can at least partially heal and integrity as well as functionality of the injured portion of the nephron be restored [22–24]. Collectively, these observations indicate that regeneration can occur to some extent in animals and humans. Indeed, the kidney contains a stem/progenitor cell system, defined as the “renopoietic system” dedicated to renal epithelial cell replacement [22]. Further, mobilization of endogenous stem cell reservoir (e.g., bone marrow) and exogenous delivery of stem cell regimens constitute promising venues of addressing diabetic complications such as nephropathy. Importantly, however, this approach must be combined with treatment modalities aimed at achieving strict control of metabolic abnormalities of the disease given the detrimental impact of hyperglycemia on stem cell number and function. This contention is supported by a recent study which investigated the impact of hyperglycemic stress on kidney stem cells which were isolated from the renal papilla showing expression of MSC markers (e.g., N-cadherin, nestin, CD133, CD29, CD90, and CD73) [25]. When these cells were co-cultured with hypoxia-injured renal tubular epithelial cells, they expressed CK18, a marker of mature epithelial cells thereby suggesting that kidney stem cells can differentiate into renal tubular epithelial cells. Importantly, however, culturing kidney stem cells in a high glucose environment impaired their differentiation ability and tolerance to hypoxia. Authors suggest that hyperglycemia compromises the reparative ability of kidney stem cells and could result in decreased ability to recover from injury. The importance of strict metabolic control is also clearly supported by observations that regression of renal lesions in diabetic subjects, following pancreatic transplantation, requires achievement of at least 5 years of normoglycemia [24].

Our observation of reduction in stem cell subsets in the peripheral blood of db/db mice is consistent with other reports indicating that EPCs are reduced in the blood of patients with type 2 diabetes compared to their controls, independent of concomitant risk factors [26]. The reduction in EPCs and associated decreased reparative capacity, in response to endothelial injury, are believed to contribute to a higher risk for cardiovascular disease associated with diabetes mellitus. Consequently, the reduction in EPCs is an important pathogenic factor contributing to microangiopathy which is intimately linked with diabetic complications including nephropathy. Aside from a reduced number, EPCs also show impaired functional features including adhesion, proliferation, and tubulogenesis. Thus, both reduced number and impaired function of EPCs compromise the ability to counter diabetes/hyperglycemia-induced injury [26]. Aside from EPCs, (bone marrow-derived) MSCs play important roles, largely via paracrine mechanism, in repair and regeneration of damaged tissues. Importantly, however, bone marrow-derived MSCs of streptozotocin-induced diabetic rats display impaired proliferation, paracrine release of various factors (e.g., vascular endothelial growth factor), anti-apoptosis, and myogenic differentiation in transplanted tissues [11]. In addition, impairment of bone marrow-derived HSCs is also a feature of diabetes thereby leading to endothelial progenitor cell dysfunction and reduced neovascularization following ischemic insult to the tissue [13].

As alluded earlier, decreased proliferation is believed to contribute to reduced number of stem cells in diabetes mellitus. We now provide evidence that increased apoptosis is also an important contributing factor to decreased number of stem cells in this condition. This is consistent with our recent study indicating marked increase in apoptotic/necrotic cell death in whole kidney cell preparations of db/db than db/m mice, an effect associated with increased GADD153, a marker of increased endoplasmic reticulum (ER) stress response [10]. Aside from ER stress response, hyperglycemia upregulates several pathways (e.g., protein kinase C, polyol pathway, advanced glycation end products, and hexosamine) which along with mitochondrial dysfunction cause increased oxidative stress [25]. In turn, oxidative stress regulates a complex web of signaling pathways which, among other effects, determine cell fate. For example, oxidative stress causes activation of a number of pro-apoptotic kinase signaling intermediates; these include several isoforms of protein kinase C, apoptosis signal-regulating kinase 1, c-Jun-N-terminal kinase and caspase, among others [27]. Thus, hyperglycemia-induced upregulation of pro-apoptotic pathways likely underlies increased cell death not only for the whole kidney as we have shown previously [10] but also in stem cell subsets in the present study in the db/db mice. However, there are very likely to be individual differences in the degree of hyperglycemia-induced upregulation in apoptosis among diabetic patients. This is evidenced by the fact that nephropathy is seen in 25 to 40 % of the diabetics [1]—implying 60 to 75 % have been able to avoid it, at least for some time. Quantitative and qualitative variations abound in biology and medicine, as do all complex adaptive systems. The increase in apoptosis may be an adaptive response to ER stress in some individuals. For these patients, the sum total cost is less to have programmed cell death and avoid spilling DAMP (damage-associated molecular pattern) which can further stimulate the inflammatory responses that are already elevated. What is needed is for us to build and iteratively improve multi-variate high-dimensional computational models based on experimental and clinical data such as provided in this report. It is only with this mechanistic approach that future therapies can be individualized based on serum marker profiles, deep understanding, and logic, rather than experience and hope.

### Outlook

The paradigm shift in health care from reactive to perspective medicine is crystallized in the concept of predictive, preventive, and personalized medicine (PPPM) [28, 29]. At the heart of PPPM is the ability to predict individuals who are at risk of developing a disease and/or its complications. With respect to type 2 diabetes, it is increasingly apparent that not all patients progress to develop end-stage renal disease, further emphasizing the need for accurate prediction of those at risk of developing this devastating complication. Nonetheless, it is abundantly clear that the diabetic milieu is detrimental to repair and reparative mechanisms that maintain organ homeostasis. Utilizing an animal model of type 2 diabetic nephropathy, we now show that the decline in the peripheral blood and renal tissue levels of stem cell subsets is associated with increased apoptosis. This observation is important for several reasons. First, since impairment of stem cells is likely to precede renal injury, early assessment and monitoring of peripheral blood stem cells could serve as a useful tool for predicting individuals who may progress to end-stage renal disease. Second, strict metabolic control could not only preserve the endogenous pool of stem cells and their repair/regenerative capacity but also improve the outcome of stem cell-based therapies. Third, although strict metabolic control is a noble objective, it is often difficult to achieve for many patients which not only jeopardizes mobilization of their endogenous repair and regenerative capacity, as it relates to function of stem cells, but also is a major impediment to harnessing the full therapeutic potential of stem cell-based therapies. Interestingly, however, it is increasingly clear that the beneficial effects of stem cells are largely related to their release of a whole host of soluble factors and their subsequent paracrine effects rather than to their transdifferentiation [30, 31]. Thus, it is plausible that patient’s own peripheral blood-derived stem cells could be procured and subjected to in vitro expansion followed by preparation of their lysates/extracts for subsequent administration, thereby bringing “true” meaning to individualized treatment approach. To that end, further pre-clinical and clinical research is essential to establish the value of this approach and determine whether it can circumvent impediments to stem cell-based therapy such as their survivability in the diabetic milieu and their homing to intended organ(s), among others [32–34]. This approach could complement other innovative or existing approaches to treatment of diabetes mellitus and its complications [35].

## Conclusion

This study shows that apoptosis of stem cells likely contributes to development of nephropathy in diabetes mellitus.
